# Increased urine IgM excretion predicts cardiovascular events in patients with type 1 diabetes nephropathy

**DOI:** 10.1186/1741-7015-7-39

**Published:** 2009-08-04

**Authors:** Rafid Tofik, Ole Torffvit, Bengt Rippe, Omran Bakoush

**Affiliations:** 1 Department of Acute Medicine, Lund University, Lund, Sweden; 2 Department of Nephrology, Lund University, Lund, Sweden

## Abstract

**Background:**

Diabetic nephropathy, a major complication of diabetes, is characterized by progressive renal injury and increased cardiovascular mortality. An increased urinary albumin excretion due dysfunction of the glomerular barrier is an early sign of diabetic nephropathy. An increased urinary excretion of higher molecular weight proteins such as IgM appears with progression of glomerular injury. We aim here to study the prognostic significance of urine IgM excretion in patients with type 1 diabetes mellitus (type 1 diabetic nephropathy).

**Methods:**

This is an observational study of 139 patients with type1 diabetes mellitus (79 males and 60 females) under routine care at the diabetic outpatient clinic at the Lund University Hospital. The median follow-up time was 18 years (1 to 22) years. Urine albumin and urine IgM concentration were measured at time of recruitment.

**Results:**

Overall 32 (14 male and 18 female) patients died in a cardiovascular event and 20 (11 male and 9 female) patients reached end-stage renal disease. Univariate analysis indicated that patient survival and renal survival were inversely associated with urine albumin excretion (RR = 2.9 and 5.8, respectively) and urine IgM excretion (RR = 4.6 and 5.7, respectively). Stratified analysis demonstrated that in patients with different degrees of albuminuria, the cardiovascular mortality rate and the incidence of end-stage renal disease was approximately three times higher in patients with increased urine IgM excretion.

**Conclusion:**

An increase in urinary IgM excretion in patients with type 1 diabetes is associated with an increased risk for cardiovascular mortality and renal failure, regardless of the degree of albuminuria.

## Background

Diabetic nephropathy (DN) develops in up to 30% of patients who have had diabetes for more than 20 years [1,2]. DN is characterized by persistent albuminuria, elevated blood pressure, and progressive decline in renal function [3]. Development of DN is associated with an increased risk of cardiovascular (CV) complications and mortality [4,5]. However, a large interindividual variation in the rate of decline in kidney function and mortality has been reported [3,6]. This highlights the need for identification of risk factors and early predictors of progression. An increased urinary albumin excretion is an early sign of DN. Impairment of the tubular protein reabsorption or in the charge-selectivity of the glomerular filtration barrier are probably the major causes of albuminuria in the early stages of type 1 DN [7]. An impairment of the glomerular size-selectivity and increased urine excretion of high molecular weight (HMW) proteins are seen in advanced stages of DN [8,9]. Increased urinary IgM excretion reflects an abundance of highly non-selective pore pathways in the glomerular filter [10]. Our studies on chronic glomerular disease generally show an association between increased urinary IgM excretion and poor kidney and patient survival [11,12]. The present study aims to evaluate the prognostic impact of increased urine IgM excretion in comparison to degree of albuminuria in an unselected population of patients with type 1 diabetes.

## Methods

In this observational follow-up study, patients with type 1 diabetes mellitus regularly attending our out-patient clinic at the Lund University Hospital were identified and recruited prospectively between 1984 and 2003. Forty-six (25 male and 21 female) patients had an albumin excretion rate in the microalbuminuric range, 48 (25 male and 23 female) had a urinary albumin excretion rate in the macroalbuminuric range, and 45 (29 male and 16 female) patients had a urinary albumin excretion rate in the normal range. The level of albuminuria was confirmed in at least two out of three consecutive urine samples. A total of 139 patients with type 1 diabetes were followed prospectively until October 2007 or death. The study was approved by the Ethics Committee at Lund University Hospital, and all patients gave informed consent. The patient characteristics are shown in Table 1. The median age was 35 years (18 to 80), and the median serum creatinine was 85 μmol/l (42 to 486). Present medications and blood pressure were taken from the patient records. Causes of death were traced from the National Death Register at the Swedish Board of Health and Welfare, and the patients' hospital records [13], Table 2. CV death was classified as all deaths where unequivocal non-CV death was not established. End-stage renal disease (ESRD) was defined as start of renal replacement therapy (dialysis or kidney transplantation) or serum creatinine >500 μmol/l.

**Table 1:** Characteristic of 139 patients with type 1 diabetes divided according to initial degree of albuminuria into normo (45), micro (46), and macro (48).

Variable	Normal	Micro	Macro	*P *value
**At baseline:**				
Sex (Male/Female)	44 (29/15)	46 (25/21)	49 (25/24)	0.3, ns
Age (years)	34 (20-72)	35 (18-80)	38 (21-79)	0.09, ns
Duration of diabetes	11 (1-54)	18 (1-65)	25 (1-67)	<0.001
S. creatinine (μmol/l)	74 (54-110)	80 (42-175)	103 (61-486)	<0.001
GFR (ml/min/1.73 m^2^)	91(45-141)	78 (28-144)	60(9-105)	<0.001
Urine IgM (mg/mmol·10^-3^)	6.7(1.7-31.8)	8.7(2.5-40)	11.5(2.8-363)	0.009
HbA1c %	7.6(4.5-13.4)	8.8(5.5-13.2)	9.1(6.2-12.7)	0.01
ACEI/ARBs, n/n (%)	0/0 (0%)	3/3 (13%)	15/5 (40.8%)	<0.001
MAP, mmhg	92(78-110)	96(80-127)	103(82-133)	<0.001
**During follow-up:**				
Follow up time, years	19(2-22)	19(2-22)	9(1-22)	0.01
MAP, mmhg	93(73-127)	99(78-125)	103(73-147)	0.005
HbA1c %	8.1(4.5-13.9)	8.0(5.0-11.1)	8.2(4.5-13.4)	0.61
ACEI/ARBs, n/n (%)	5/0 (11.4%)	9/15 (53.3%)	20/16 (76.6%)	<0.001
CV-Mortality rate per patient-year	0.003994674	0.0110957	0.035836177	<0.001
Renal failure rate per patient-year	0	0.007407	0.032895	<0.001

Abbreviations: ACEI: Angiotensin converting enzyme inhibitors; ARBs: Angiotensin II receptor antagonists; CV: cardiovascular; ESRD: End stage renal disease; GFR: glomerular filtration rate; HbA1c: glycosylated hemoglobin; MAP: Mean arterial blood pressure.

**Table 2:** Causes of death of 38 patients died during a median of 18 years follow-up time of 139 patients with type 1 diabetes mellitus.

Cause		No. (M/F)
Cardiovascular		32 (14/18)
	Cardiac arrest	8 (3/5)
	MI	12 (5/7)
	Heart failure	3 (2/1)
	Stroke	9 (4/5)

Malignancy		2(0/2)
	Lung cancer	1 (0/1)
	Uterus cancer	1 (0/1)

Sepsis		2(0/2)

Acute hypoglycemia		1(1/0)

Suicide		1(0/1)

Total		38(15/23)

Abbreviations: MI = Myocardial Infarction.

### Laboratory measurements

Glycosylated hemoglobin (HbA1c) was analyzed by high-performance liquid chromatography. Serum and urinary creatinine concentrations were analyzed by an enzymatic method (creatinine-amidino-hydrolase; KODAK EKTACHEM analyser, Instrument Kodak, NY, USA). Urinary albumin concentration was measured on fresh urine samples using turbidimetry (Cobas Mira S, Roche). The detection limits was 5 mg/l, and the coefficient of variation was between 1% and 8%. Urine samples were kept at -20°C until analysis for IgM in December 2006. Urine IgM concentrations were measured by ELISA as described elsewhere [10]. The lower detection limit for the urine IgM assay is 1 μg/l; the intra-assay and inter-assay variation is 4.6% and 10.9%, respectively. Glomerular filtration rate (GFR) was estimated using the MDRD formula [14,15], where eGFR = {186.3 × (serum creatinine mg/dl)^-1.154 ^× (age)^-0.203 ^(× 0.742 if female).

### Statistical analysis

Data are presented as median (range) or mean ± SE. The patients groups were compared with non-parametric statistical tests Mann-Whitney U and Kruskal-Wallis H tests. The Fisher's exact test and Pearson Chi square (χ^2^) test were used when appropriate. Incidence rate of ESRD and mortality rate were calculated per person-year. Kaplan-Meier curves were used for survival analysis. Univariate Cox regression analysis was used to examine the baseline variables predictive of CV mortality and ESRD. Results described as relative ratios (RR) and the 95% confidence intervals (95% CI). Due to sample size restriction, subsequent stratified analysis was conducted only to compare the prognostic effect of albuminuria and IgM-uria. The patients were divided into low and high IgM excretion groups according to the median value (0.01 mg/mmol) of urine IgM excretion. Microalbuminuria was defined as a urinary albumin excretion rate of 3 to 20 mg/mmol; <3 mg/mmol was regarded as normal and >20 mg/mmol was regarded as macroalbuminuria [16]. Statistical analyses were performed using statistical package SPSS 13.0 (SPSS, Chicago Illinois, USA). The level of statistical significance was set at *P *values < 0.05.

## Results

The median time to death or end of follow up by October 2007 was 18 years (1 to 22). During the follow up 38 patients died and 20 (11 male and 9 female) patients developed ESRD. Six (1 male and 5 female) patients died from non-CV events and 32 (14 male and 18 female) patients in a CV event (Table 2). These 32 (14 male and 18 female) patients were included in a further analysis of CV-mortality.

Univariate Cox-regression analysis identified the following predictors of CV mortality: age (HR = 11.5, 95% CI 3.5 to 38, *P *< 0.001), duration of diabetes (HR = 7.8, 95% CI 3 to 20, *P *< 0.001), mean arterial blood pressure (HR = 3.6, 95% CI 1.6 to 8.1, *P *= 0.002), serum creatinine (HR = 3.3, 95% CI 1.5 to 7.3, *P *= 0.004), eGFR (HR = 9.3, 95% CI 3.3 to 27.0, *P *< 0.001), urinary albumin excretion (HR = 2.9, 95% CI 1.7 to 5.0, *P *< 0.001) and urinary IgM excretion (HR = 4.6, 95% CI 2.0 to 10.7, *P *< 0.001). The predictors of ESRD were: duration of diabetes (HR = 4.3, 95% CI 1.6 to 12, *P *=0.005), mean arterial blood pressure (HR = 3.9, 95% CI 1.4 to 10.8, *P *= 0.009), serum creatinine (HR = 23.3, 95% CI 3.1 to 174, *P *= 0.002), GFR (HR = 12.4, 95% CI 2.9 to 54.0, *P *= 0.001), urinary albumin excretion (HR = 5.8, 95% CI 2.4 to 14, *P *< 0.001) and urinary IgM excretion (HR = 5.7, 95% CI 1.9 to 17, *P *= 0.002). Stepwise multivariate Cox regression analysis shows that urine IgM excretion, independently of the level of albuminuria, predicts CV mortality and renal failure (*P *= 0.004). There was no significant difference in CV mortality between those treated and those not treated by ACE inhibitors, (29.2% vs. 15.5%, *P *= 0.054) or between those treated by ACEi or ARBs (35.1% vs. 22.6, *P *= 0.26).

### Patient and renal outcome by degree of albuminuria

Table 1, shows the baseline and follow-up data of the 139 patients studied divided according to the degree of baseline albuminuria. As expected, the duration of diabetes, serum creatinine and blood pressure were significantly higher in the macroalbuminuric group. During follow-up there was no difference in HbA1c, and more patients with macroalbuminuria received ACE inhibitors.

#### Cardiovascular mortality

Three patients in the normo-albuminuric and eight patients in the microalbuminuric group died of a CV event during the follow-up time, compared with 21 (36.8%) patients in the macroalbuminuric group (*P *< 0.001). The risk of CV mortality was 3.6% per patient-year in patients with macroalbuminuria compared with approximately 1.1% in patients with microalbuminuria; that is, the RR increment was 3.23 (Table 1).

#### Renal survival

Fifteen (30.6%) patients from the macroalbuminuric group developed ESRD compared with only five (5.6%) patients in the microalbuminuric group (*P *< 0.001). The risk of ESRD was approximately 3.3% per patient-year in patients with macroalbuminuria compared with approximately 0.7% in patients with microalbuminuria (RR 4.44, Table 1).

### Patient and renal outcome by degree of IgM-uria

Table 3 shows the baseline and follow-up data of the 139 patients studied divided according to baseline urine IgM excretion. Patients with increased urine IgM excretion had significantly increased urine albumin excretion, higher blood pressure and higher serum creatinine compared with patients with low urine IgM excretion. The low IgM group had a mean urine IgM concentration of 0.0055 ± 0.0017 mg/mmol, and the high IgM group had a mean urine IgM concentration of 0.0258 ± 0.0457 mg/mmol. There was no difference in follow-up glycemic control between IgM groups, and patients with high IgM excretion had a higher percentage of renoprotective treatment with ACE inhibitors.

**Table 3:** Characteristic of 139 patients with type 1 diabetes divided according to the median urine IgM level into low (<0.01 mg/mol) and high IgM (>0.01 mg/mol) groups.

Variable	Low IgM group	High IgM group	*P *value
**At baseline:**			
Sex (male/female)	71 (49/22)	68 (30/38)	0.003
Age (years)	35(18-74)	37(20-80)	0.05
Duration of diabetes (yr)	15 (1-65)	24 (1-67)	0.001
HbA1c %	8.3 (4.5-13.4)	9 (5.5-13.2)	0.06, ns
Albuminuria (mg/mmol)	3.6(0.23-268)	21.3 (0.26-640)	<0.001
S. Creatinine μmol/l	78 (54-216)	86 (42-486)	0.009
GFR (ml/min/1.73 m^2^)	88 (27-141)	67(9-144)	<0.001
MAP mmhg	93 (80-120)	100 (78-133)	0.004
ACEI/ARBs, n/n (%)	3/2 (7.1%)	15/6 (31%)	<0.001
**During follow-up:**			
Follow up time (years)	19 (2-22)	14 (1-22)	<0.001
MAP mmhg	97 (73-119)	103 (73-147)	0.038
HbA1c (%)	8.25 (4.5-13.9)	8 (4.5-13.4)	
0.7, ns			
Albuminuria (mg/mmol)	1.3 (0.1-676)	20.5 (0.1-1101)	<0.001
ACEI/ARBs, n/n (%)	12/14 (37%)	22/17 (59%)	0.008
CV-Mortality rate per patient-year	0.005838	0.029104	<0.001
Renal failure rate per patient-year	0.003451	0.022161	0.003

Abbreviations: ACEI: Angiotensin converting enzyme inhibitors; ARBs: Angiotensin II receptor antagonists; CV: cardiovascular; ESRD: End stage renal disease; GFR: glomerular filtration rate; HbA1c: glycosylated hemoglobin; MAP: Mean arterial blood pressure.

#### Cardiovascular mortality

Seven (9.9%) patients with low urinary IgM excretion died from CV events during the follow-up time compared with 25 (36.8%) patients with high urinary IgM excretion (*P *< 0.001). The risk of CV mortality was 2.9% per patient-year in patients with high urine IgM excretion compared with approximately 0.6% in patients with low in IgM excretion (RR = 4.98, Table 3).

#### Renal survival

Sixteen (23.5%) patients from the high IgM group developed ESRD during the follow-up period compared with only four (5.6%) patients with low urinary IgM excretion (*P *= 0.003). The risk of ESRD was 2.2% per patient-year in patients with high urinary IgM excretion compared with approximately 0.35% in patients with low in IgM excretion (RR = 6.4, Table 3).

### Patient and renal outcome by degree of albuminuria and IgM-uria

#### Cardiovascular mortality

**Figure 1:**
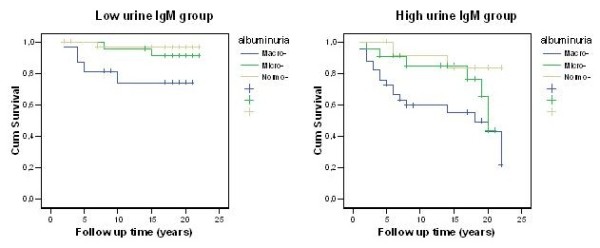
**Cardiovascular mortality rate by albuminuria and urine IgM**.

Patients with microalbuminuria and high urine IgM excretion had 2.1% per patient-year risk for CV mortality, compared with approximately 0.46% in those with low IgM excretion (RR = 4.6, Table 4). Patients with macroalbuminuria and high urine IgM excretion had 4.8% per patient-year risk for CV mortality, compared with 1.7% in those with low IgM excretion, (RR = 2.8, Table 4). Even patients with normoalbuminuria and high urine IgM excretion had significantly higher risk for CV mortality compared with those with low IgM excretion (0.9% vs. 0.57% per patient-year, RR = 1.6, Table 4). The pattern of cumulative patient survival by degree of albuminuria and IgM-uria are show in Figure 1.

**Table 4:** Patient and renal outcome of 139 patients with type 1 diabetes by degree of albuminuria and IgM-uria.

Stage of DN	Baseline urine IgM excretion	Rate of renal failure per patient-year	CV-Mortality rate per patient-year	10 year Patient survival	10 year Renal survival
Normoalbuminuria (*n *= 44)	Low (*n *= 31)	0	0.005671	96.5%	100%
	High (*n *= 13)	0	0.009009	91.7%	100%
Microalbuminuria (*n *= 46)	Low (*n *= 24)	0.004750594	0.004577	95.7%	95.6%
	High (*n *= 22)	0.011811024	0.021127	84.9%	87.5%
Macroalbuminuria (*n *= 49)	Low(*n *= 16)	0.014354067	0.017167	73.9%	84%
	High (*n *= 33)	0.052631579	0.048159	59.5%	53%

#### Renal survival

**Figure 2:**
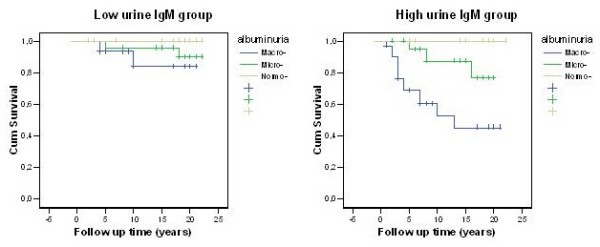
**Renal survival rate by albuminuria and IgM-uria**.

Patients with microalbuminuria and high urine IgM excretion had approximately 1.2% per patient-year risk for renal failure, compared with approximately 0.48 in those with low IgM excretion (RR = 2.4, Table 4). Patients with macroalbuminuria and high urine IgM excretion had 5.3% per patient-year risk for renal failure, compared with 1.4% in those with low IgM excretion, (RR = 3.7, Table 4). The pattern of cumulative renal survival by degree of albuminuria and IgM-uria is show in Figure 2.

## Discussion

In this cohort of long-time follow-up (median 18 years) of patients with type 1 diabetes, with and without diabetic nephropathy, we found that patients with an increased urinary IgM excretion had a higher mortality from CV causes, and higher disease progression rate to ESRD compared with patients with low urinary IgM excretion. In accordance with other reports, this study demonstrates that CV death due to atherosclerosis is the major cause of mortality in diabetic patients. Furthermore, the mortality was strongly associated with duration of diabetes mellitus and the degree of albuminuria [3,4]. To our knowledge our study is the first one investigating the impact of an increased urine IgM excretion on DN disease progression in type 1 diabetic patients.

We were able to show that the association of urine IgM excretion with a higher risk for CV mortality and ESRD is largely independent of the level of albuminuria. Patients with DN and increased urine IgM excretion, either in the micro or the macroalbuminuric stage, had at least a threefold higher risk of CV mortality and renal failure than those with low urinary IgM excretion, (Table 4, Figures 1 and 2).

Previous studies suggest that the development of DN in patients with type 1 diabetes is due to an early impairment of charge selectivity of the glomerular filter. The size selectivity dysfunction appears later, along with progression of the disease [17]. Thus, albuminuria *per se *could be due to impairment of glomerular charge or size selectivity, that is, could reflect minor or severe glomerular damage [18,19]. IgG-uria (molecular radius 55 Å), studied previously by others, is mainly due to an increased transport through the large pores [17]. However, the very large protein measured in this study, IgM (molecular radius 120 Å), is able to pass the glomerular filtration barrier only through very large defects (shunts) [10]. Thus, the occurrence of IgM in the final urine reflects a markedly increased population of highly unselective glomerular pathways and probably a more severe glomerular damage [11,12].

An increase in renal vascular resistance, due to atherosclerotic vascular disease, may induce ischemia and more severe structural changes in the glomeruli, and cause an increase in urinary excretion of HMW proteins through such shunt-like pathways [20-23]. This may explain why urinary IgM excretion correlates with CV events in this cohort of type 1 diabetic patients.

## Conclusion

While albuminuria is an early predictor of DN progression, we were able in this study to show that an increased urinary IgM excretion in patients with DN was an independent predictor of CV complications. However, further studies are needed to evaluate the correlation between urine IgM excretion and surrogate markers of atherosclerosis in patients with CV disease and diabetes mellitus.

## Competing interests

The authors declare that they have no competing interests.

## Authors' contributions

RT undertook concept and design of the study, data collection, analysis and interpretation of data, and drafting of the manuscript. OT contributed to study design, data collection, interpretation of data, and critical review of intellectual content. BR contributed to interpretation of data, and critical review of intellectual content. OB contributed to the concept and design of the study, analysis and interpretation of data, drafting the manuscript, and critical review of intellectual content. All authors read and approved the final manuscript.

## Pre-publication history

The pre-publication history for this paper can be accessed here:


